# Diagnosis and treatment delay of head and neck cancers during COVID-19 era in a tertiary care academic hospital: what should we expect?

**DOI:** 10.1007/s00405-021-06834-1

**Published:** 2021-04-30

**Authors:** Pietro De Luca, Antonella Bisogno, Vito Colacurcio, Pasquale Marra, Claudia Cassandro, Angelo Camaioni, Ettore Cassandro, Alfonso Scarpa

**Affiliations:** 1 Department of Medicine, Surgery and Dentistry, University of Salerno, Salerno, Italy; 2 Department of Surgical Sciences, University of Turin, Turin, Italy; 3 Otolaryngology Department, San Giovanni-Addolorata Hospital, Rome, Italy

**Keywords:** COVID-19, Diagnosis delay, Head and neck cancer, Oncology, Lockdown

## Abstract

**Background:**

Since the spreading of SARS-CoV-2 from China, all deferrable medical activities have been suspended, to redirect resources for the management of COVID patients. The goal of this retrospective study was to investigate the impact of COVID-19 on head and neck cancers’ diagnosis in our Academic Hospital.

**Methods:**

A retrospective analysis of patients treated for head and neck cancers between March 12 and November 1, 2020 was carried out, and we compared these data with the diagnoses of the same periods of the 5 previous years.

**Results:**

47 patients were included in this study. We observed a significative reduction in comparison with the same period of the previous 5 years.

**Conclusions:**

Our findings suggest that the COVID-19 pandemic is associated with a decrease in the number of new H&N cancers diagnoses, and a substantial diagnostic delay can be attributable to COVID-19 control measures.

## Introduction

Since February 2020, the coronavirus disease COVID-19 has spread from Wuhan (China) as a worldwide public health emergency [1], and Italy has been one of the most affected countries from the beginning of the pandemic [2]. The first confirmed Italian case of COVID-19 was documented on February 21, 2020, from Codogno (Lombardy) [3], although a recent study showed the presence of SARS-CoV-2 in wastewater earlier than that date, simultaneously in different regions, changing the understanding of the spread of the virus in Italy [4]. The local and national health authorities put in place various containment strategies, as national and local lockdown, to keep under control the spread of the virus, trying to protect patients and health-care workers. On March 12, 2020, all deferrable clinical and surgical activities have been suspended, to redirect resources for the management of COVID patients [5], as a part of the national strategy to flatten the curve of the SARS-CoV-2 infections and reduce the impact on the Italian National Health System. After that, our Otolaryngology Department ceased all eligible activity, with a substantial reduction of daily surgical and outpatient activity. Even if non-elective clinical and surgical activities continued to take place during the pandemic, the fear of contagion could have discouraged patients, in particular older patients and those with pre-existing pathologies [6].

When Regional Government resumed elective activity at the end of Italy’s lockdown, we observed an increase in the diagnosis of head and neck malignancies, probably reflecting delayed access to health-care services for patients with symptoms suspected of being cancer-related. In addition to that, we could expect a certain number of patients still not seeking medical examination for their symptoms, especially if mild and nonspecific, in wait for the contagion rate to drop. Moreover, in April 2020, at the peak of COVID-19 pandemic, Italian screening rates for colon, breast, prostate, and lung were significantly lower than baseline rates of the previous year [7]. As reported by other authors [8, 9], the delay in the diagnosis and treatment of tumors during this pandemic could represent a dramatic effect of the current situation.

The goal of this retrospective study was to investigate the impact of COVID-19 pandemic on head and neck cancer diagnosis in our tertiary care Academic Hospital.

## Methods

A retrospective analysis of all patients diagnosed and surgically treated for head and neck cancers at the Division of Otorhinolaryngology of the University Hospital “San Giovanni di Dio e Ruggi D’Aragona” (Salerno, Italy) between March 12, 2020 [the beginning of sanitary lockdown in our Region (Campania)] and November 1, 2020, was carried out (Group 2020). A comparison of the number of diagnosis and surgical intervention performed during this period was carried out with the same periods of time of the 5 previous years (Group 2019, Group 2018, Group 2017, Group 2016, Group 2015), to evaluate the effective impact of COVID-19 pandemic in terms of delay in the diagnosis and treatment of head and neck tumors. Extracted data included population size and characteristics (age range, gender), type of tumor, and total number of each type of tumor per year.

Moreover, for the 2020 group, a telephone survey was carried out by a single ENT resident (Pietro De Luca) to assess the mean time between symptoms onset and medical referral, and how much the fear of contagion and the critical situation of the health-care services impacted on that delay. The patients were asked to specify the time of the onset of symptomatology; the date of the first otolaryngology consultation was extracted from our database. Moreover, respondents were asked “Have you delayed or avoided medical care due to concerns related to COVID-19?”, and to quantify (months) the eventual delay.

## Results

**Fig. 1:**
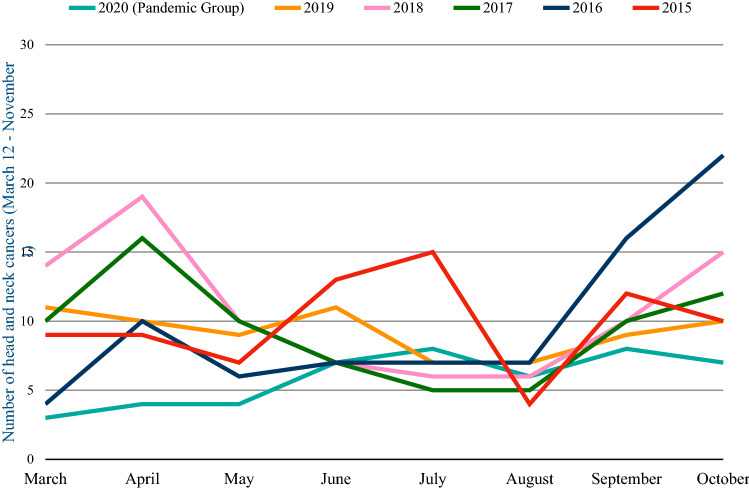
Comparison between the number of diagnoses of head and neck cancers at our University Hospital in the last 5 years and in the pandemic period of COVID-19 (March 1, 2020—November 1, 2020)

**Fig. 2:**
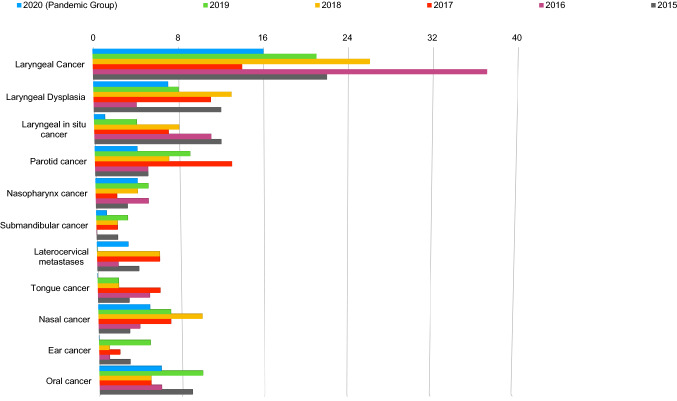
Comparison between the number of diagnoses of each type of head and neck tumor provided in the last 5 years and in the pandemic period of COVID-19 (March 12, 2020—November 1, 2020)

To investigate the potential impact of COVID-19 pandemic on the diagnosis and treatment of head and neck cancers at “San Giovanni di Dio e Ruggi D’Aragona” University Hospital (Salerno, Italy), this retrospective study compared the number of new cancer diagnoses between March, 12 and November 1, 2020 with the same periods of the previous 5 years. A total of 47 patients newly diagnosed with head and neck cancers newly were included in this study, as shown in Table 1 and in Figs. 1 and 2. We observed a significative reduction in comparison with the same period of the previous 5 years (− 36.5% if compared with 2019; − 44% if compared to 2018; − 37.3% if compared to 2017; − 41.2% if compared to 2016; − 40.5% if compared to 2015). The most relevant reduction rate was observed between March 12rd, 2020 and May 1st, 2020. Therefore, taking number of new diagnoses between March 12 and November 1 2020, and comparing it with the mean number of diagnoses made in the same period of the previous 5 years, we observed only 59.5% of the expected cases. Patients were aged 27–92 years and the mean age at the diagnosis was 65.1 years (67 for men, 61.1 for women); the prevalence of men was 68% (Table 2).

**Table 1:** General characteristics of the population

	March 12, 2020–November 1, 2020	March 12, 2019–November 1, 2019	March 12, 2018–November 1, 2018
Head and neck oncological total surgical procedures, n	47	74	84
Gender (male/female), n	32/15	49/25	60/24
Mean age (range), y	65.1 (27–92)	63.7 (29–87)	64.2 (18–91)
Men mean age (range), y	67 (41–92)	64.5 (29–87)	65 (35–91)
Women mean age (range), y	61.1 (24–87)	61.1 (35–84)	62.5 (18–82)

	March 12, 2017–November 1, 2017	March 12, 2016–November 1, 2016	March 12, 2015–November 1, 2015
Head & Neck Total Surgical Procedures, n	75	80	79
Gender (male/female), n	59/16	59 / 21	53 / 26
Mean age (range), y	65.4 (31–96)	58.6 (14–87)	61.7 (6–83)
Men mean age (range), y	65.2 (31–96)	64.6 (25–87)	64.8 (6–83)
Women mean age (range), y	66.2 (51–90)	60.38 (40–84)	61.1 (37–83)

**Table 2:** Comparison between the number of diagnoses of each type of head and neck cancer in the last 5 years and in the pandemic period of COVID-19 (March 1, 2020—November 1, 2020)

Type of tumor	March 12—November 1, 2020	March 12—November 1, 2019	March 12—November 1, 2018	March 12—November 1 2017	March 12—November 1 2016	March 12—November 1, 2015
Laryngeal cancer	16 (34%)	21 (28.4%)	26 (30.9%)	14 (18.6%)	37 (46.2%)	22 (27.8%)
Laryngeal dysplasia	7 (14.9%)	8 (10.8%)	13 (15.5%)	11 (14.7%)	4 (5%)	12 (15.2%)
Laryngeal in situ cancer	1 (2.1%)	4 (5.4%)	8 (9.5%)	7 (9.3%)	11 (13.7%)	13 (16.4%)
Parotid cancer	4 (8.5%)	9 (12.1%)	7 (8.3%)	13 (17.3%)	5 (6.2%)	5 (6.3%)
Nasopharynx cancer	4 (8.5%)	5 (6.7%)	4 (4.7%)	2 (2.7%)	5 (6.2%)	3 (3.8%)
Submandibular gland cancer	1 (2%)	3 (4%)	2 (2.3%)	2 (2.7%)	0	2 (2.5%)
Laterocervical metastases	3 (6.4%)	0	6 (7.1%)	6 (8%)	2 (2.5%)	4 (5%)
Tongue cancer	0	2 (2.7%)	2 (2.3%)	6 (8%)	5 (6.2%)	3 (3.8%)
Nasal cancer	5 (10.6%)	7 (9.4%)	10 (11.9%)	7 (9.3%)	4 (5%)	3 (3.8%)
Ear cancer	0	5 (6.7%)	1 (1.2%)	2 (2.7%)	1 (1.2%)	3 (3.8%)
Oral cancer	6 (12.7%)	10 (13.5%)	5 (5.9%)	5 (6.7%)	6 (7.5%)	9 (11.4%)

We then analyzed the reduction in diagnostic rate for the different head and neck cancers, comparing the number of new diagnoses for each kind of tumors in 2020 with the mean number of diagnoses of the same tumor for the previous 5 years. The malignancies that showed the greater reduction were laryngeal cancer (16 vs 24), in situ laryngeal cancer (1 vs 8.6), parotid cancer (4 vs 7.8), and tongue cancer (0 vs 3.6).

As an additional evaluation, a telephone survey for the patients of 2020 group was conducted; we were able to collect data only from 52 patients (88%). Of them, 43 patients (82.7%) delayed specialist evaluation because of concerns about COVID-19, and people with multiple underlying medical conditions (*n* = 34, 79%), such as diabetes or hypertension, were significantly more likely to avoid or delay urgent or emergency care compared to people without these conditions. The mean total delay from the onset of symptoms to the ENT evaluation was 5.3 months (0.7—8.2, months).

## Discussion

Since the outbreak of COVID-19 in Wuhan (China), our National Healthcare System has been profoundly stressed and forced to reallocate resources and health-care workers to manage COVID-19 patients. From March 12, 2020, the Italian lockdown has been associated with a substantial decrease in most of non-COVID-19 services [10]**,** with a dramatic effect on the patient group requiring time-critical access to health-care services. Although restriction of social life has been effective in reducing the number of COVID-19 infections [11], it also may have a negative impact on the diagnosis of cancers. We also should consider that a delay in the diagnosis of head and neck cancers during COVID-19 pandemic may thus result in an increase in cancer-related mortality [12]. According to our results, there was a significant decrease in the number of new head and neck cancer diagnoses between March 12, 2020 and November 1, 2020, compared with the same period of the 5 previous years. The findings of this retrospective study are in line with the literature. Several publications have already reported the delay in the diagnosis of cancer patients during COVID-19 pandemic. A study from Jacob et al. showed that the COVID-19 pandemic had a significant negative impact on cancer diagnosis in Germany, especially for those tumors for which national screening program was suspended [13]. A study using data of Dutch Cancer Registry showed a decrease of 26–60% in the number of new cancer diagnoses in the period between April 6, 2020 and April 12, 2020 [14].

In addition, Yang et al. reported that 32.5% of patients with nasopharyngeal carcinoma, during the waiting period of radiotherapy, received an additional cycle of chemotherapy to the original treatment strategy, increasing unnecessary treatment intensity of patients [15]. In our study, no patient received unnecessary radio-chemioterapic treatment during the waiting period of surgery. A hypothesis that may explain the fall in the number of new cancer diagnoses could be that the fear of going to hospital, seen as a possible source of infection, and the fear to be at risk of contagion, especially for older patients, patients with mild symptoms, and those with serious comorbidities, can be considered as primary factor. This is confirmed by an Italian study from Vanni et al. which reported how women with breast lesion and those with diagnosis of breast cancer refused surgery because of the risk of contagion [16]. Furthermore, patients are usually referred by their general practitioners to otolaryngology practice; instead, during COVID-19 pandemic, telephone consultations have largely replaced face-to-face consultations in primary care, making it difficult for a general practitioner to understand the origin and the severity of some symptoms.

To the best of our knowledge, in our country, no research has yet been performed analyzing the effects of COVID-19 pandemic on head and neck cancer diagnosis and treatment. Moreover, no other study compared the number of diagnoses made in 2020 with the diagnoses made in the previous 5 years. Nevertheless, the study results should be interpreted in the light of several limitations. First, quantifying the impact of delays in diagnosis on stage and prognosis is complex. Furthermore, potential effect of delays in head and neck cancer diagnosis and treatment on prognosis may vary with the type of cancer; currently, no work investigated the association between delay and prognosis in patients with head and neck cancer. The real impact of this delay for head and neck malignancies will be probably more clear in the next months and years, and more studies will be required to adequately analyze it.

## Conclusion

Our findings suggest that the COVID-19 pandemic is associated with a decrease in the number of new head and neck cancers diagnoses in our Academic Hospital, and a substantial diagnostic delay can be attributable to COVID-19 lockdown and to SARS-CoV-2 control measures. Currently, we are not able to predict how much this could affect the prognosis of these patients, and this reflects the need for policy interventions, especially in case of future lockdown, to contain additional cancer deaths resulting from delay in diagnosis. Given that the COVID-19 pandemic is still ongoing in Italy, measures should be taken to improve head and neck cancer diagnosis, and patients should be educated to understand the risk of COVID-19 contagion versus the risk of not seeking health-care advice, especially patients experiencing symptoms suggestive of head and neck tumors.
